# Comparing the Appetitive Learning Performance of Six European Honeybee Subspecies in a Common Apiary

**DOI:** 10.3390/insects12090768

**Published:** 2021-08-27

**Authors:** Ricarda Scheiner, Kayun Lim, Marina D. Meixner, Martin S. Gabel

**Affiliations:** 1 Behavioral Physiology & Sociobiology, Biocenter, University of Würzburg, Am Hubland, 97074 Würzburg, Germany; kayunlim@snu.ac.kr (K.L.); martinsebastian.gabel@llh.hessen.de (M.S.G.); 2 Laboratory of Insect Biosystematics, Department of Agricultural Biotechnology, Seoul National University, Seoul 08826, Korea; 3 Landesbetrieb Landwirtschaft Hessen, Bee Institute Kirchhain, Erlenstraße 9, 35274 Kirchhain, Germany; marina.meixner@llh.hessen.de

**Keywords:** adaptation, *Apis mellifera*, olfactory learning, proboscis extension response, sucrose responsiveness, genetic diversity

## Abstract

**Simple Summary:**

This study is the first to compare the associative learning performance of six honeybee subspecies from different European regions in a common apiary. We quantified sucrose responsiveness prior to appetitive olfactory proboscis extension learning to dissociate effects of motivation and cognition. Our results show that *Apis mellifera iberiensis* displayed a significantly poorer learning performance compared to other *Apis* subspecies from across Europe, which did not differ from each other. Possible causes are discussed.

**Abstract:**

The Western honeybee (*Apis mellifera* L.) is one of the most widespread insects with numerous subspecies in its native range. How far adaptation to local habitats has affected the cognitive skills of the different subspecies is an intriguing question that we investigate in this study. Naturally mated queens of the following five subspecies from different parts of Europe were transferred to Southern Germany: *A. m. iberiensis* from Portugal, *A. m. mellifera* from Belgium, *A. m. macedonica* from Greece, *A. m. ligustica* from Italy, and *A. m. ruttneri* from Malta. We also included the local subspecies *A. m. carnica* in our study. New colonies were built up in a common apiary where the respective queens were introduced. Worker offspring from the different subspecies were compared in classical olfactory learning performance using the proboscis extension response. Prior to conditioning, we measured individual sucrose responsiveness to investigate whether possible differences in learning performances were due to differential responsiveness to the sugar water reward. Most subspecies did not differ in their appetitive learning performance. However, foragers of the Iberian honeybee, *A. m. iberiensis*, performed significantly more poorly, despite having a similar sucrose responsiveness. We discuss possible causes for the poor performance of the Iberian honeybees, which may have been shaped by adaptation to the local habitat.

## 1. Introduction

The natural range of the Western honeybee (*A. mellifera*) expands throughout Africa, Europe, Western and Central Asia to Western China in the East [1,2,3,4,5,6,7]. The intraspecific diversity of *Apis mellifera* is remarkable, with currently about 30 subspecies described [1,7,8,9,10]. The different subspecies display diverse adaptations to a wide variety of geographic areas and environmental factors. They can be grouped into five evolutionary lineages (A, M, C, O, and Y), based on morphometric and molecular studies [7,8,9,11]. Whereas the subspecies of lineage A are spread across Africa, those belonging to lineage Y originate in North-Eastern Africa. Subspecies of lineage M are distributed widely in Western and Northern Europe, while lineage C comprises subspecies originating from South-Eastern Europe. Lineage O stems from the Near and Middle East. However, the native distribution of the different subspecies has been altered gravely by human interference and the current situation no longer represents the original distribution [5,9,12]. The worldwide demand for highly profitable honeybee colonies and the breeding efforts focusing on certain behavioral traits such as low aggressiveness has promoted the introduction of subspecies to locations outside their natural range, causing accidental and deliberate hybridization and leading to the jeopardization of native populations of *A. mellifera* subspecies.

Selection has not only shaped the morphology of the different subspecies but also behavioral traits such as defensiveness (e.g., breeding for low aggressiveness) and annual colony development cycles (e.g., early spring development in the economically popular *A. m. carnica* [2,3,5,7,13,14,15]). However, huge behavioral differences have not only been observed between different subspecies of honeybees, but also among members of the same colony. A good example is individual responsiveness to sucrose, which has frequently been employed as a general indicator of the physiological state of a honeybee [16,17,18]. It differs grossly between individuals performing different social tasks [19,20,21,22,23,24], and between seasons [25]. Importantly, it allows us to make predictions about the appetitive learning performance of the individual, because it correlates positively with cognitive performance in appetitive associative learning [23,24,25,26,27]. The more responsive a honeybee is to sucrose, the higher is her learning score, i.e., the better is her cognitive performance. So far, all of these experiments have been performed with workers of two subspecies of the Western honeybee, i.e., *A. m. carnica* and *A. m. ligustica.* Whether this relationship between cognitive performance and sensory responsiveness to sucrose is universal for different honeybee subspecies is an intriguing question. Similarly, it is an exciting open question whether different honeybee subspecies share similar cognitive capacities.

**Figure 1:**
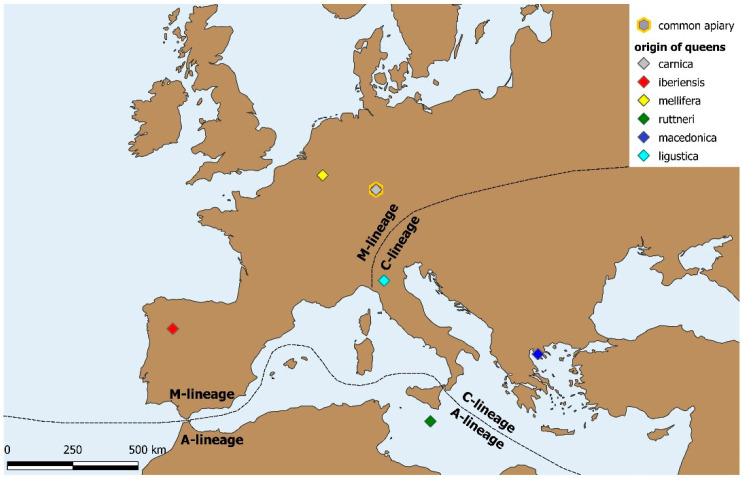
Map of lineage distribution ranges and points of origin of the different honeybee subspecies studied.

We here studied for the first time the appetitive olfactory learning performance in different subspecies of *Apis mellifera* maintained in a common garden apiary. We performed the experiments with colonies of six different European subspecies of *A. mellifera*, covering three of the five known evolutionary lineages: *A. m. mellifera* and *A. m. iberiensis* of lineage M, *A. m. carnica*, *A. m. macedonica*, and *A. m. ligustica* from lineage C, and *A. m. ruttneri* from lineage A (Figure 1). We hypothesized that the different subspecies should only differ in their appetitive learning performance if they differed in their responsiveness to sucrose, because this factor is an important determinant of learning performance [16,19,23,24,27]. Sucrose responsiveness might be shaped by local adaptation to climate and habitat. Secondly, we aimed to investigate whether the correlation between individual sucrose responsiveness and appetitive learning performance described for *A. m. carnica* and *A. m. ligustica* is also present in the other subspecies, pointing towards a general rule for appetitive associative learning.

The Carniolan bee *A. m. carnica* (Figure 1), a representative of lineage C, is native to South-Eastern Austria and the North-Western Balkan Peninsula. It is currently one of the most frequently used subspecies in commercial beekeeping and bee science worldwide. From the late 19th century on, hives have been exported from the Westernmost tip of its native distribution area to various destinations [7]. The “grey Carniolan bee” has become very popular in Germany, because of its gentle temperament and low swarming tendency, important characteristics for beekeeping. Nowadays, the Carniolan bee, *A. m. ligustica*, and the commercial hybrid Buckfast have by far the widest distribution area, often threatening other subspecies.

The *A. m. iberiensis* bees used in our experiment came from Bragança in Northern Portugal (Figure 1). The natural distribution of this subspecies covers the whole Iberian Peninsula and the Balearic Islands [7,14,28,29], where it partially overlaps with an imported commercial stock of other subspecies [5]. Ruttner [7] describes different ecotypes adapted to both cold and warm climate conditions. Brood rearing is highly economical without wasting resources and has good overall potential for applied beekeeping. This subspecies is further known for using high quantities of propolis, displaying a nervous behavior on the combs, and being ferociously defensive [7,15].

The original distribution of *A. m. mellifera* (lineage M) extended throughout central Europe North of the Alps including the United Kingdom, Ireland, and Scandinavia in the North, all over France in the West, and across Poland to the Ural mountain range in the East [7]. Today, it has been replaced by other subspecies in large parts of its former distribution area [5,13,30,31,32]. This includes wide parts of Germany, where Carniolan stock (*A. m. carnica*) is now predominant. Various ecotypes have been described which share a brooding rhythm adapted to nectar flow, ample use of propolis, and a nervous behavior on the combs with a tendency to defensiveness [7,15]. Our *A. m. mellifera* bees stem from a breeding apiary in Belgium (Figure 1).

The subspecies *Apis mellifera ruttneri* (lineage A) is endemic to the archipelago of Malta, which constitutes its former and actual range of distribution (Figure 1; [14,33]). Due to its small area of distribution and frequent imports of other commercially used subspecies, *A. m. ruttneri* is highly prone to genetic introgression [14,33]. Its brood cycle is adjusted to seasonal nectar flows and xeric conditions, while colonies show a moderate use of propolis and sometimes fierce defensive behavior [33]. While the distinct defensive behavior, especially under hot and dry weather conditions, is an unfavorable trait for their use in apiculture, *A. m. ruttneri* can cope with predatory wasps and the challenging seasons of the respective habitat [33].

The natural distribution range of *A. m. macedonica* (lineage C) extends from Northern Greece across North Macedonia, Bulgaria, Romania, and Moldova to Southern Ukraine in the North [2,7]. Ruttner [7] describes *A. m. macedonica* as gentle but sometimes inclined to swarm and susceptible to Nosemosis. He also reports ample use of propolis and brood reduction as a reaction to unsuitable (especially hot and dry) weather conditions. Our bees were derived from Northern Greece (Figure 1).

The original distribution range of *A. m. ligustica* (lineage C) covers the Apennine Peninsula [7]. In recent times, however, *A. m. ligustica* has been spread around the globe for its beekeeping value. Apart from *A. m. carnica*, this subspecies is by far the most commonly used in commercial apiculture [5,13]. Beekeepers appreciate its high fertility, gentleness, little use of propolis, and high productivity under good foraging conditions [7,15]. Under poor foraging conditions or in cold climates, however, colonies of *A. m. ligustica* do not adjust their brood rearing: a trait resulting in lower honey yields for beekeepers and/or starvation of colonies through accelerated store consumption [7,15]. Our *A. m. ligustica* bees originated in Northern Italy (Figure 1).

Here we asked whether and in how far selection for traits important for commercial beekeeping and natural adaptation to local climate and habitat have shaped sensory and cognitive capacities of the different honeybee subspecies.

## 2. Materials and Methods

### 2.1. Bees and Hive Management

**Figure 2:**
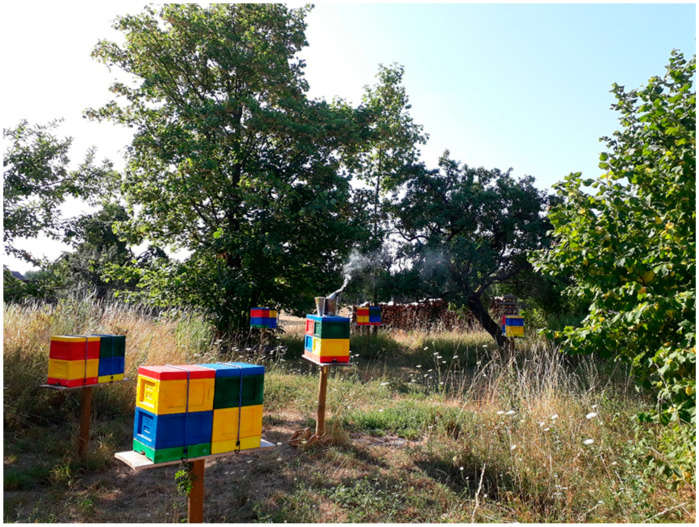
Location of experimental hives in a common apiary with four colonies of five subspecies of the Western honeybee *Apis mellifera* located in Southern Germany.

Experiments were conducted in the summer of 2018 at the University of Würzburg (Bavaria, Germany). The experimental bees were derived from 23 queen-right colonies of six different subspecies of the Western honeybee *(Apis mellifera*). The colonies were headed by open mated queens from *A. m. carnica*, *A. m. ligustica*, *A. m. macedonica*, *A. m. mellifera*, *A. m. ruttneri*, or *A. m. iberiensis* (*n* = 3–4 resp.). The queens were derived from populations central to the current distribution area of their respective subspecies (Figure 1) and introduced into shook swarms of *A. m. carnica* at least ten weeks prior to data collection. All queens had been shipped according to valid veterinary legislation and were registered in the TRACES-Database. To prevent genetic pollution of the local honeybee population, all hives were equipped with excluder grids at the entrance, allowing only worker bees to fly freely. In addition, all drone brood was removed from the colonies, and the tip of one wing of each queen was clipped following the protocol of Human et al. [34]. Hive stands were grouped according to subspecies to avoid drifting of homecoming forager bees between subspecies. For the same reason, only one pair of two differently colored hives from one subspecies was placed on each hive stand with opposite flying directions (Figure 2). Despite the occurrence of some drought periods, the foraging conditions were comparatively good throughout the experimental period, with rich floral nectar flows and honeydew.

### 2.2. Harnessing of Bees

Returning foragers were collected at the hive entrance. Only foragers without pollen in their corbiculae were selected, assuming that they were nectar foragers. All bees were caught between morning and midday. Bees were caught individually in small glass vials, they were immobilized on ice and mounted in small brass tubes according to the standard protocol of Scheiner et al. [16]. Mounted bees were fed with 5 μL of a 30% sucrose solution (according to Matsumoto et al. [35]) and placed in a dark incubator (temperature 35 °C, relative humidity 70%) for one hour to recover from anesthesia and for sufficient motivation to learn [19,21,22,23,24,25,26,27,36].

### 2.3. Sucrose Responsiveness

Sucrose responsiveness was tested prior to training as described elsewhere [16,22,23,36,37]. Briefly, both antennae of each bee were sequentially stimulated with water and a series of sucrose concentrations (0.1%, 0.3%, 1.0%, 3.0%, 10%, 30% *w*/*v*) in ascending order. The inter-trial interval was two min to prevent intrinsic sensitization [36]. It was recorded which sucrose concentration elicited the proboscis extension response (PER) for each bee. The total number of proboscis extension responses is the gustatory response score (GRS) of a bee [16].

### 2.4. Appetitive Olfactory Learning and Memory Tests

To quantify associative olfactory learning we used a standard protocol for classical conditioning with one odor described elsewhere [16,21]. Bees are required to associate the odor with a sugar-water reward. Only bees not displaying a spontaneous response to the conditioned odor were used. Bees were trained with 30% sucrose solution as unconditioned stimulus and reward and 5 µL of 1-hexanol (Sigma Aldrich, Steinheim, Germany) as the conditioned stimulus. During training, each bee was placed in a constant air stream. We delivered 5 mL of the odor/air mixture manually to the antennae of each bee. The bee experienced the odor for 1 s before the PER was elicited by touching the antennae with a 30% sucrose droplet. As soon as the bee extended her proboscis, she was fed with a small droplet of sucrose solution for 1 s. The odor was removed approximately 0.5 s after the onset of feeding, so that conditioned odor and sucrose reward overlapped in time. The inter-trial interval was 5 min. During each training trial, we recorded whether the bee displayed the conditioned PER before this response was elicited by applying sucrose to the antennae [16,21]. The total number of conditioned PERs of a bee during the 6 training trials constitutes her acquisition or learning score [16,37]. The following number of bees were conditioned: n*_carnica_*: 86, n*_iberiensis_*: 56, n*_mellifera_*: 71; n*_ruttneri_*: 74; n*_macedonica_*: 45; n*_ligustica_*: 18.

### 2.5. Statistics

All statistical analyses were conducted using SPSS Statistics 26 (IBM, Armonk, NY, USA). The GRS and acquisition scores were tested for normal distribution using the Shapiro-Wilk test. Since both scores were not distributed normally within each subspecies, non-parametric Kruskal Wallis H tests were performed with post hoc tests using *p* values corrected for multiple comparisons. To test for effects of the colony on acquisition scores we also performed Kruskal–Wallis H tests within each subspecies. The number of bees showing the correct response during the acquisition phase (i.e., acquisition curves) was analyzed with generalized estimation equations (GEE) using the binary responses in each acquisition trial as dependent variable and subspecies and GRS as factors. Sidak tests were used as post hoc tests. Correlation analyses between GRS and acquisition scores were performed using Spearman rank correlation.

## 3. Results

**Figure 3:**
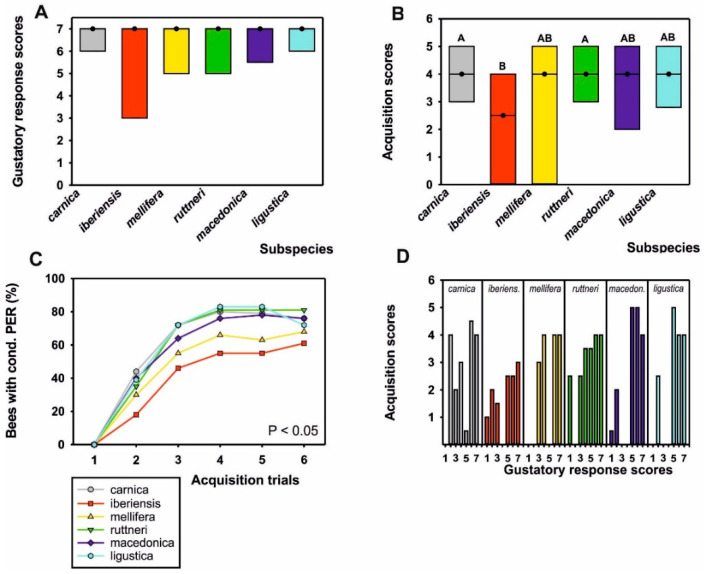
(**A**) Sucrose responsiveness measured as gustatory response scores (GRS) of foragers belonging to the six different honeybee subspecies trained in this study. Median GRS (dots) and quartiles (25%: lower line, 75%: upper line) are presented. Subspecies were all highly responsive to sucrose and did not differ in their GRS (*p* > 0.05). (**B**) Median acquisition scores (dots) of bees from subspecies trained in classical olfactory conditioning and quartiles (see **A**). Subspecies that differed significantly have different letters (for details see Table S2). (**C**) Acquisition curves of subspecies based on individual responses to the conditioned odor in each of the 6 training trials. There was a significant effect of subspecies on learning performance (*p* < 0.05), with *A. m. iberiensis* foragers performing significantly more poorly than those of *A. m. carnica* and *A. m. ruttneri* (for details see Table S3). (**D**) Correlation between GRS and acquisition scores in each subspecies. Median acquisition scores are shown for bees in each GRS class. Different subspecies are indicated by colors. Generally, the higher the GRS, the higher was the acquisition score, demonstrating a better learning performance. For the number of bees tested see Table S1.

Gustatory response scores (GRS) of bees trained to 1-hexanol were overall very high (Figure 3A) and did not differ between subspecies (*p* > 0.05, Kruskal–Wallis H Test), which indicated high responsiveness to sucrose. Based on earlier experiments with *A. m. carnica* and *A. m. ligustica* we, therefore, expected high acquisition scores in all subspecies. We pooled data from foragers from different hives within each subspecies, because the colony did not have an effect on acquisition score within each subspecies (*p* > 0.05, Kruskal–Wallis H test).

Comparison of acquisition scores yielded a significant effect of subspecies on learning performance (Figure 3B; *p* < 0.01, Kruskal–Wallis H Test). Foragers of the *A. m. iberiensis* subspecies displayed significantly lower acquisition scores than those of the *A. m. carnica* and *A. m. ruttneri* subspecies (*p* < 0.01; Table S2). For a more detailed analysis of learning behavior, we compared the learning curves of the different subspecies and included GRS as a within-subject factor in the model. Subspecies had a significant effect on learning curves (Figure 3C; *χ*^2^_(5,349)_ = 12.75, *p* < 0.05, GEE). *A. m. iberiensis* foragers performed significantly more poorly than *A. m. carnica* foragers (*p* < 0.01; Table S3) and *A. m. ruttneri* foragers (*p* < 0.01, Table S3), while all of the other subspecies did not differ in their learning curve. GRS had a large and significant effect on learning curves (*χ*^2^_(6,349)_ = 36.87; *p* < 0.001). Bees with higher GRS performed better than bees with lower GRS across subspecies. We further tested for a correlation between GRS and acquisition scores within each subspecies.

GRS correlated with acquisition scores significantly positively in the subspecies *A. m. carnica* (*ρ* = 0.34, *p* < 0.001), *A. m. mellifera* (*ρ* = 0.40, *p* < 0.001) and *A. m. ruttneri* (*ρ* = 0.32, *p* < 0.01) but not in the other subspecies (Figure 3D; *p* > 0.05, Spearman rank correlations). However, the same trend is observable in the other subspecies and it appears that the unequal distribution of GRS and partially a low sample size are related to the absence of a significant correlation.

## 4. Discussion

This is the first study comparing the cognitive abilities of six different *Apis mellifera* subspecies from across Europe under standardized conditions in a common apiary. The bees tested for their appetitive olfactory learning performance were all highly motivated, i.e., displaying a similarly high sucrose responsiveness, which is an indicator of their “learning motivation” and can predict learning performance to a high degree [19,24,27,38]. If the correlation between GRS and acquisition scores frequently demonstrated in *A. m. carnica* [24,27] and *A. m. ligustica* [19,23] were also present in the other honeybee subspecies, there should be few differences in the learning performance of the different subspecies, since the majority of differences in appetitive olfactory learning can be attributed to differences in the sucrose responsiveness of the bees [27]. In fact, most of the European honeybee subspecies we analyzed did not differ in their learning performance from each other, supporting this hypothesis. However, *A. m. iberiensis*, a subspecies native to Southern Europe, performed significantly more poorly in our classical olfactory learning paradigm compared to *A. m. carnica* and *A. m. ruttneri* and displayed a non-significant tendency to perform less well than the other subspecies. This led us to question the general nature of the correlation between GRS and acquisition scores in a simple classical olfactory conditioning assay. We found a significant and expected positive correlation in *A. m. carnica*, *A. m. mellifera*, and *A. m. ruttneri*. The reason why we did not see this correlation in *A. m. ligustica* might be due to the lower sample size in this experiment, but we nevertheless decided to include those data for comparison, and earlier experiments demonstrated a significant positive correlation between GRS and acquisition scores in this subspecies, too [19,23]. In fact, the trend for a positive correlation between GRS and acquisition scores is observable in most subspecies tested (Figure 3D), but the distribution of GRS was sometimes suboptimal for correlation analyses because most bees were highly responsive. Here, further experiments with larger samples sizes within each subspecies can help to ultimately show this correlation in all subspecies.

Learning differences between different strains of honeybees (*A. m. ligustica*) selected for high or low amounts of stored pollen [39], for example, could be explained by a different sucrose responsiveness, which correlated with the probability to collect pollen or nectar [19]. However, sucrose responsiveness did not differ between subspecies in the current experiment, and we only tested nectar foragers (Figure 3A). Thus we can largely exclude the possibility that *A. m. iberiensis* foragers were simply “less motivated” to learn compared to the *A. m. carnica* or *A. m. ruttneri* foragers. Therefore, the lower learning performance of this subspecies might be related to other factors including genetic differences related to foraging behavior. The differences in the learning performance of *A. m. iberiensis* foragers and foragers of the other subspecies appear not to be linked to the lineage of this subspecies, because *A. m. mellifera*, which is the second representative of lineage M, performs as well as the other subspecies.

It is conceivable that a differential adaptation to temperature-related stress may be linked to different learning performances. Iberian bees might have a higher energy demand to cope with heat stress and/or higher foraging activity compared to honeybees from Central Europe, similar to what has been suggested by Iqbal et al. [40] for Arabian honeybees. A greater foraging activity, in turn, leads to the accumulation of oxidative stress [41] and reduces learning performance [42,43]. However, *A. m. ruttneri* faces similar climatic stress because of high temperatures. Nevertheless, it performed significantly better than *A. m. iberiensis*. It is, therefore, more likely that a combination of factors contributes to the lower learning performance of *A. m. iberiensis* compared to *A. m. carnica* and *A. m. ruttneri*, possibly including a differential responsiveness to odors, which could further contribute to their lower learning performance, as discussed elsewhere [27].

Differential foraging strategies and related learning performance might be a further reason for a differential performance of *A. m. carnica* and *A. m. iberiensis*. Diverse mechanisms involving learning appear to be important in decision-making of individual foragers (e.g., [44]). Pérez-Claudio et al. [45] discussed the lower learning ability of *A. m. syriaca* compared to *A. m. caucasia* in reverse association task in light of a higher predation rate in their native habitat. This might favor a risk-minimizing foraging strategy with higher floral fidelity. Conversely, *A. m. caucasia* showed higher flexibility in a reverse association learning task, consistent with a previously described lower floral fidelity [10] and a lower predation rate in their foraging range [45]. Whether the foraging strategy of *A. m. carnica, A. m. ruttneri,* and the other subspecies differ from that of *A. m. iberiensis* is currently being investigated in our lab.

In an olfactory PER learning experiment comparing the performance of the Africanized honeybees in North America (*Apis mellifera scutellata* hybrid) with that of honeybees of European origin (*A. m. carnica*), the former performed significantly more poorly [46]. The authors suggested the hypothesis that the Africanized honeybee might have been ecologically more successful than the Western honeybee, which might have been related to their lower learning performance. The authors suggest that if learning did not induce additional costs, there should be universal selection for high learning performance [46]. However, a high degree of variation is maintained in natural populations of insects [47], suggesting that learning could theoretically incur a fitness cost [48,49,50]. Experiments in the fruit fly *Drosophila melanogaster* demonstrate an evolutionary trade-off between learning ability and competitive ability, supporting the hypothesis that selection for improved learning is consistently linked to a decreased competitive ability for limited food resources in larvae [51]. A similar trade-off between improved learning performance and successful, aggressive strategies is conceivable for the honeybee. Africanized honeybees are notorious for their highly defensive behavior. Similarly, the Iberian honeybee is typically more defensive than the Carniolan honeybee [7,15], which was also apparent in our apiary, where all of the subspecies were hosted under equal climatic conditions.

A recent study comparing the learning performance of *A. m. carnica*, *A. m. ligustica*, and *A. m. jemenitica* showed that similar to our findings, the former two did not differ in their PER learning performance, whereas *A. m. jemenitica* performed less well [40]. In their experiments, the smaller body size of *A. m. jemenitica* was considered a possible reason for poorer learning performance, based on studies of body size and learning performance in bumblebees [52]. However, it is debatable whether brain size is an appropriate indicator of learning behavior because there are different outcomes of experiments trying to link this factor with behavioral repertoires and cognitive abilities in animals [46,53,54]. Further, it would not explain our differences between *A. m. iberiensis* and *A. m. carnica*, since they have a similar size [7]. In addition, *A. m. ruttneri* is slightly smaller than either *A. m. carnica* or *A. m. iberiensis*. If size did matter, we would expect learning differences here, too.

An intriguing hypothesis that awaits further investigation is that *A. m. iberiensis* bees differ from *A. m. carnica* bees, *A. m. ruttneri* bees and the other subspecies tested in their amount of neurotransmitters in the brain. A higher baseline brain titer of the biogenic amine octopamine, which itself is involved in the mediation of the reward in appetitive PER learning [55], might be responsible for better learning performance. In support of this hypothesis, we could show that bees performing different social tasks (i.e., nurse bees vs. foragers or pollen collectors vs. nectar collectors), do not only differ in their titers of octopamine and its metabolic precursor tyramine [20,21,22] but also in their appetitive learning capability [21,22,24]. The appetitive learning performance of honeybees can be improved by treating the bees with octopamine [56] or tyramine [21]. Differential octopamine brain titers may be related to different foraging strategies of the different races and also to aggression. This interesting neuroecological question awaits further study. In addition, other neuromodulators such as neuropeptide F might be involved in the learning differences between *A. m. iberiensis* and the other subspecies. Studies in fruit flies show that artificial activation of neuropeptide F neurons inhibits appetitive olfactory learning by modulating the sugar reward signal during acquisition [57]. Future experiments should correlate the activity and amount of this peptide in good and poor learners of the different subspecies to test for a similar function of this peptide in honeybees. Further, *A. m. iberiensis* foragers may have a reduced size of the mushroom bodies, important brain centers involved in learning and multimodal processing, or may have fewer synapses in their calyces of the mushroom bodies, leading to reduced appetitive learning performance. This question has to be studied in future experiments.

## 5. Conclusions

The most significant finding of our study is that differences in cognitive abilities are part of the intraspecific diversity in *A. mellifera*, similar to what has been demonstrated for other behavioral traits [2,3,58,59,60]. While most of the subspecies tested here were very similar in associative learning capacities and the correlation between sucrose responsiveness and appetitive learning performance, the Iberian honeybee surprised with a reduced learning performance, independent of the main motivational factor sucrose responsiveness. It could be linked to different foraging strategies, the higher aggressiveness of the Iberian bees, different amounts of neurotransmitters in the brain, different sizes of brain neuropils important for learning, or to genetic differences, all of which might have been shaped by ecological factors. It is an exciting open question how the neuroecology of foraging behavior and learning might thus be interlinked and shaped by adaptation to local climate and habitat.

## Data Availability

Data will be uploaded to Dryad.

## Supplementary Materials

https://www.mdpi.com/article/10.3390/insects12090768/s1, Table S1: Number of bees in each GRS class of each subspecies, Table S2: Comparison of acquisition scores. Test statistics for Kruskal–Wallis H tests (KW) and *p* values corrected for multiple comparisons are given, Table S3: Comparison of acquisition curves. Test statistics for Sidak post hoc tests are given.
