# Supplementary material for: Comparing the Appetitive Learning Performance of Six European Honeybee Subspecies in a Common Apiary
Source: Insects. 2021 Aug 27;12(9):768. doi: 10.3390/insects12090768 (PMC8468525; doi:10.3390/insects12090768)
Supplement: Supplementary file 1 [file insects-12-00768-s001.zip › insects-1322938-supplementary.pdf]

insects MDPI

Supplementary Materials:

Table S1. Number of bees in each GRS class of each subspecies.

GRS Class	*carnica*	*iberiensis*	*mellifera*	*ruttneri*	*macedonica*	*ligustica*	Total
**1**	3	5	5	2	4	0	19
**2**	4	5	7	2	3	2	23
**3**	6	6	3	6	1	0	22
**4**	3	0	2	4	0	0	9
**5**	2	4	3	6	3	1	19
**6**	8	4	14	3	4	5	38
**7**	60	32	37	51	30	10	220
**total**	86	56	71	74	45	18	350

Table S2. Comparison of acquisition scores. Test statistics for Kruskal Wallis H tests (KW) and p values corrected for multiple comparisons are given.

	carnica	*iberiensis*	*mellifera*	*ruttneri*	*macedonica*	*ligustica*
carnica	********	KW = 3.74<br>***p* = 0.003**	KW = 2.19<br>*p* = 0.43	KW = 0.21<br>*p* = 1.00	KW = 0.39<br>*p* = 1.00	KW = 0.24<br>*p* = 1.00
iberiensis	********	********	KW = 1.63<br>*p* = 1.00	KW = 3.44<br>***p* = 0.009**	KW =2.86<br>*p* = 0.49	KW =2.14<br>*p* = 0.49
mellifera	********	********	********	KW =1.91<br>*p* = 0.85	KW =1.47<br>*p* = 1.00	KW = 1.09<br>*p* = 1.00
ruttneri	********	********	********	********	KW = 0.20<br>*p* = 1.00	KW = 0.11<br>*p* = 1.00
macedonica	********	********	********	********	********	KW = 0.03<br>*p* = 1.00
ligustica	********	********	********	********	********	********

Table S3. Comparison of acquisition curves. Test statistics for Sidak post hoc tests are given.

	carnica	*iberiensis*	*mellifera*	*ruttneri*	*macedonica*	*ligustica*
carnica	********	***p* = 0.003**	*p* = 0.29	*p* = 1.00	*p* = 1.00	*p* = 1.00
iberiensis	********	********	*p* = 0.95	***p* = 0.005**	*p* = 0.13	*p* = 0.13
mellifera	********	********	********	*p* = 0.35	*p* = 1.00	*p* = 0.84
ruttneri	********	********	********	********	*p* = 1.00	*p* = 1.00
macedonica	********	********	********	********	********	*p* = 1.00
ligustica	********	********	********	********	********	********
